# Divergent Desires: Sexual Expression in ASD and ADHD Individuals

**DOI:** 10.1192/j.eurpsy.2025.2319

**Published:** 2025-08-26

**Authors:** C. Pinheiro Ramos, A. F. Reis, M. J. Freire, S. Mendes

**Affiliations:** 1 Setúbal Hospital Center, Setúbal, Portugal

## Abstract

**Introduction:**

Autism Spectrum Disorder (ASD) and Attention-Deficit/Hyperactivity Disorder (ADHD) can both impact psychosexual functioning.

ASD individuals may struggle with appropriate sexual interactions due to challenges in social communication, understanding social norms, and sensory sensitivities. Restricted and repetitive behaviors may also lead to a focus on specific sexualized behaviors.

ADHD-related symptoms may impact psychosexual functioning. Impulsivity can lead to risky sexual behavior, while inattention may increase the risk of sexual dysfunction. Additionally, prescribed ADHD medication has been found to disrupt sexual functioning.

**Objectives:**

Our aim is to better understand the psychosexual profile of neurodivergent individuals.

**Methods:**

A narrative review was carried out using various databases, including PubMed.

**Results:**

Literature suggests a higher prevalence of sexual ambivalence, as well as increased homosexual, bisexual, and asexual tendencies among ASD-individuals. They may also exhibit more inappropriate sexual behavior, which can put them at risk of legal consequences.

Research suggests that ADHD-individuals may have a higher frequency of homosexual experiences and females with ADHD report greater ambivalence about their gender identity. ADHD is associated with risky sexual behaviors, but ADHD-medications may have a protective effect against early pregnancy and STIs.

**Conclusions:**

The literature suggests that individuals with ADHD and/or ASD can lead to different psychosexual functioning compared to neurotypical peers.

Further research is needed to understand the contributing factors.

**Disclosure of Interest:**

None Declared

